# A narrative review on the role of hesperidin on metabolic parameters, liver enzymes, and inflammatory markers in nonalcoholic fatty liver disease

**DOI:** 10.1002/fsn3.3729

**Published:** 2023-10-08

**Authors:** Nava Morshedzadeh, Amirhossein Ramezani Ahmadi, Vahideh Behrouz, Elias Mir

**Affiliations:** ^1^ Student Research Committee Kerman University of Medical Sciences Kerman Iran; ^2^ Department of Nutrition, Faculty of Public Health Kerman University of Medical Sciences Kerman Iran; ^3^ Isfahan Endocrine and Metabolism Research Center Isfahan University of Medical Sciences Isfahan Iran

**Keywords:** fatty liver, hesperidin, inflammation, nonalcoholic, obesity

## Abstract

Insulin resistance, oxidative stress, hyperlipidemia, and inflammation play main roles in the development of nonalcoholic fatty liver disease (NAFLD). Some studies have reported that hesperidin can reduce hyperglycemia and hyperlipidemia by inhibiting inflammatory pathways. In the current study, our purpose was to evaluate whether it can influence the primary parameters in NAFLD and improve the treatment effectiveness for future trials. Various studies have found that hesperidin involves multiple signaling pathways such as cell proliferation, lipid and glucose metabolism, insulin resistance, oxidative stress, and inflammation, which can potentially affect NAFLD development and prognosis. Recent findings indicate that hesperidin also regulates key enzymes and may affect the severity of liver fibrosis. Hesperidin inhibits reactive oxygen species production that potentially interferes with the activation of transcription factors like nuclear factor‐κB. Appropriate adherence to hesperidin may be a promising approach to modulate inflammatory pathways, metabolic indices, hepatic steatosis, and liver injury.

## Graphical abstract

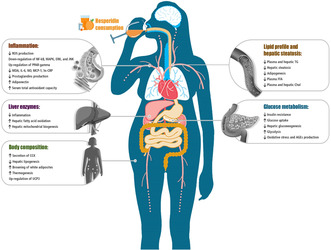

## 1 INTRODUCTION

Nonalcoholic fatty liver disease (NAFLD) has reached epidemic proportions globally. It is estimated that more than 14%–24% of the general adult population worldwide has NAFLD, and of these, more than 80% have clinical morbid obesity with NAFLD (Browning et al., 2004; Youssef & McCullough, 2002). NAFLD is the consequence of a prolonged disorder caused by the agglomeration of triglycerides within the hepatocytes, which is characterized by hepatic steatosis (fat accumulation in the liver) without significant evidence of inflammation. Nonalcoholic steatohepatitis (NASH) occurs when it develops to the next stage, hepatic steatosis, with the initiation of an inflammatory process, eventually leading to liver damage, fibrosis, and cirrhosis (Clark & Diehl, 2003; Sporea et al., 2018). NAFLD is regarded as a multifactorial condition that can be affected by lifestyle, cultural, environmental, genetic, sedentary lifestyle, hypercaloric diets, and physiological and metabolic factors. Interestingly, the consumption of Western diets, especially high consumption of simple sugars and saturated fats, increases the prevalence of obesity and its associated problems, such as hyperlipemia, insulin resistance, and NAFLD among others (Arab et al., 2018; Behrouz & Yari, 2022; Jalilvand et al., 2020; Karimzadeh et al., 2022). Scientific attention is focused on the implementation of revolutionary and helpful solutions for the management of this disease and its comorbidities (Bateni et al., 2022; Jafari & Behrouz, 2023).

Hesperidin is a glycosidic flavanone present generally in citrus fruits like sweet oranges and lemons. It, as a citrus flavonoid, has biological and pharmacological attributes including anticarcinogenic, anti‐inflammatory, antioxidative, vascular protective, and lipid‐decreasing activities (Parhiz et al., 2015). It seems that hesperidin can enhance hypercholesterolemia and fatty liver by persuading fatty liver (steatosis) degeneration, inhibiting cholesterol synthesis and absorption, and moderating mRNA expression of lipid metabolism‐related enzymes (Wang et al., 2011). On the other hand, studies showed that hesperidin was able to ameliorate insulin resistance by modulating oxidative stress signaling pathways (Tian et al., 2021). Actually, hesperidin can perform beneficial and therapeutic impacts on the fatty liver due to several mechanisms including decreasing serum glucose concentration, hepatic lipid levels, fatty acid oxidation, and hepatic steatosis induced by inflammation (Jung et al., 2006). Moreover, evidence has suggested its role in inhibiting tumor cell metastasis, and improving metabolic components, and diabetes complications.

Notwithstanding the considerable interest in phytochemicals, to the best of our knowledge, the effects of hesperidin and probable mechanisms of action in NAFLD have not been discussed in the review literature. For this review, a literature search of Scopus, PubMed, Web of Science, and EMBASE was completed up to January 22, 2023, by the following terms: “NAFLD,” “NASH,” “nonalcoholic fatty liver disease,” “nonalcoholic steatohepatitis,” “lipid profile,” “hypercholesterolemic,” “hypertension,” “glucose metabolism,” “insulin resistance,” “diabetes,” “hyperlipidemia,” “body composition,” “inflammation,” “inflammatory factors,” combined with “hesperidin.” The present review highlights the ongoing development to identify the targets of hesperidin in liver diseases and the possible pathways influenced by hesperidin in NAFLD by reviewing all in vitro studies, human studies, and animal models relevant to the purpose of the present review.

## 2 HESPERIDIN

Hesperidin, as a derivative of dihydro flavonoids, is widely present in legumes, birch, oleander, Rutaceae, and citrus plants (especially juice) such as lemon, orange, lime, and grapefruit. There may also be Lamiaceae (mint family), honeybush (*Cyclopia maculata*), and aromatized tea (Hajialyani et al., 2019). Hesperidin (3,5,7 trihydroxy flavanone 7‐rhamnoglucoside, C_28_H_34_O_15_), also known as hesperetin 7‐rutinoside or 7‐O‐glycoside hesperitin, a flavonoid glycoside that was first extracted from citrus peel by the French chemist Lebreton (lemon peel, etc.; Roohbakhsh et al., 2015). The hesperidin‐chitooligosaccharide complex, which is obtained from the reaction of hesperidin with chitooligosaccharide, increases the solubility in water, which improves its antioxidant activity (Xiong et al., 2019).

Hesperidin has anti‐inflammatory features, but it also has low water solubility and, like many other flavonoids, is poorly absorbed in the small intestine. In general, the bioavailability of hesperidin is limited because of its low solubility in water and its being affected by the intestinal microbiota. However, the amount of hesperidin is higher in the layer of the peel and albedo (a white soft middle layer part) and seeds of citrus fruits (Aruoma et al., 2012; Chen, Wang, et al., 2020).

The biochemical properties of hesperidin remain for 2 years if stored at −20°C. Oral or topical administration or injection of hesperidin derivatives in the mice model did not show any side effects (Pyrzynska, 2022). The potential benefits of hesperidin in improving type 2 diabetes, cardiovascular disease, hypertension, glucose metabolism, lipid profile, cancer, and neurodegenerative disorders have been extensively investigated throughout recent decades (Man et al., 2019).

## 3 HESPERIDIN AND LIPID PROFILE

Investigating the effects of hesperidin on NAFLD is not possible without considering the effects of this compound on lipid profile, glucose homeostasis, body composition, and inflammation (Table 1). The possible hypolipidemic effects of hesperidin against hypercholesterolemia were investigated in some studies (Li et al., 2022; Monforte et al., 1995; Wang et al., 2011). One study examined the effects of consuming hesperidin on indices of lipid parameters in subjects with type 2 diabetes. The results have shown that hesperidin supplementation decreased the plasma level of total cholesterol (TC), although no alterations were observed in triglyceride (TG), high‐density lipoprotein cholesterol (HDL‐C), and low‐density lipoprotein cholesterol (LDL‐C; Eghtesadi et al., 2016). Considering that the liver stashes lipids in the form of triglycerides, liver fat aggregation is correlated with increased lipotoxicity due to high levels of free fatty acids, cholesterol, and other lipid metabolites in NAFLD (Schweitzer & Finck, 2014). In a rodent model study, hesperidin decreased hepatic steatosis, adipose tissue, serum TC, and retinol‐binding protein (RBP)‐4 density in rats fed a high‐fat diet, although the reduction in LDL‐C and TG concentration was not significant after hesperidin administration. In addition, hesperidin may ameliorate hypercholesterolemia and fatty liver by downregulating mRNA expression of RBP, heart fatty acid–binding protein (H‐FABP), and cutaneous fatty acid–binding protein (C‐FABP; Wang et al., 2011). Similarly, another study has shown that lipid‐related factors such as H‐FABP and C‐FABP which play key roles in fatty acid metabolism were improved by hesperidin supplementation (Qian et al., 2016). The results of another study conducted on rats with a high‐cholesterol diet showed that diosmin‐hesperidin significantly improved HDL‐C levels, but did not alter other lipid parameters (Yasım et al., 2011).

**TABLE 1:** Randomized controlled trials that evaluated the effects of hesperidin on metabolic parameters, liver function, and inflammation.

References	Condition (*n*)	Treatment	Duration	Main results
Eghtesadi et al. (2016)	T2DM (45)	Intervention group: 500 mg/day hesperidin (*n* = 23) Control group: 500 mg/day placebo (*n* = 22)	8 weeks	A significant decrease in FBG and HbA1c A significant increase in serum insulin within hesperidin group A significant decrease in TC within hesperidin group No significant changes in inflammatory factors
Cheraghpour et al. (2019)	NAFLD (50)	Intervention group: 1‐g/day hesperidin (*n* = 25) Control group: 1‐g/day placebo (*n* = 25)	12 weeks	A significant decrease in ALT, GGT, TC, and TG A significant decrease in hepatic steatosis, hs‐CRP, TNF‐α, and NF‐κB
Haidari et al. (2015)	Myocardial infarction (75)	Intervention group: 600 mg/day hesperidin (*n* = 38) Control group: 600 mg/day placebo (*n* = 37)	4 weeks	A significant decrease in E‐selectin A significant increase in adiponectin and HDL‐C A significant decrease in IL‐6, hs‐CRP, leptin, and other lipid profile within hesperidin group
Demonty et al. (2010)	Patients with hypercholesterolemia (204)	Intervention group 1: 800 mg/day hesperidin (*n* = 59) Intervention group 2: 500 mg/day naringin (*n* = 64) Control group: placebo (*n* = 65)	4 weeks	No significant changes in TC, LDL‐C, HDL‐C, and TG
Ribeiro et al. (2017)	Patients with obesity (78)	Intervention group: 500 mL/day orange juice (*n* = 39) Control group: placebo (*n* = 39)	12 weeks	A significant decrease in insulin, HOMA‐IR, TC, LDL‐C, and hs‐CRP
Lima et al. (2019)	Healthy individuals (10)	Intervention group: 300 mL/day orange juice (*n* = 10) No control group	8 weeks	A significant decrease in TC, LDL‐C, glucose, TG, and HOMA‐IR A significant increase in HDL‐C No significant changes in body composition
Homayouni et al. (2017)	T2DM (64)	Intervention group: 500 mg/day hesperidin (*n* = 31) Control group: placebo (*n* = 29)	6 weeks	A significant increase in TAC within hesperidin group A significant decrease in serum fructosamine, 8‐OHDG, and MDA within hesperidin group No significant changes in FBG and insulin resistance
Morand et al. (2011)	Healthy people with overweight (24)	Intervention group 1: 500 mL/day orange juice (*n* = 24) Intervention group 2: 500 mL/day control drink plus hesperidin (*n* = 24) Control group: 500 mL/day control drink plus placebo (*n* = 24)	4 weeks	No significant changes in the inflammatory factors (CRP, IL‐6, VCAM‐1, and ICAM‐1) No significant changes in TC, LDL‐C, glucose, TG, insulinemia
Constans et al. (2015)	Subjects with cardiovascular risk factors (25)	Intervention group: 600 mL orange juice (*n* = 25) Control group: control drink (*n* = 25)	4 weeks	A significant increase in apoA‐I and apoB within the intervention group No significant changes in glucose, TG, hs‐CRP, and fibrinogen
Yari et al. (2021)	NAFLD (100)	Intervention group 1: LMP + 30 g/day flaxseed (*n* = 25) Intervention group 2: LMP + 1 g/day hesperidin (*n* = 25) Intervention group 3: LMP + combination of 30 g flaxseed and 1 g hesperidin (flax‐hes) (*n* = 25) Control group: LMP + placebo (*n* = 25)	12 weeks	A significant decrease in ALT, FBS, insulin resistance, insulin sensitivity, and fatty liver index
Hanawa et al. (2008)	Healthy subjects with moderately high BMI (75)	Intervention group 1: 500 mg/day G‐hesperidin (*n* = 15) Intervention group 2: 500 mg/day G‐hesperidin with 25 mg/day caffeine (*n* = 15) Intervention group 3: 500 mg/day G‐hesperidin with 50 mg/day caffeine (*n* = 15) Intervention group 4: 500 mg/day G‐hesperidin with 75 mg/day caffeine (*n* = 15) Control group: placebo (*n* = 15)	12 weeks	A significant decrease in BMI in the G‐hesperidin with 75‐mg caffeine group compared to the placebo No significant change in TG
Aptekmann and Cesar (2010)	Middle‐aged women with overweight (26)	Intervention group: 500 mL/day orange juice (*n* = 13) Control group: placebo (*n* = 13)	12 weeks	A significant increase in HDL‐C within the intervention group A significant decrease in LDL‐C within the intervention group A significant decrease in fat mass and weight within the intervention group
Yari et al. (2020)	Metabolic syndrome (49)	Intervention group: 500 mg/day hesperidin (*n* = 25) Control group: placebo (*n* = 24)	12 weeks	A significant decrease in FBG, TG, and TNF‐α A significant decrease in glucose, insulin, TG, TC, LDL‐C, TNF‐α, and hs‐CRP within hesperidin group
Homayouni et al. (2018)	T2DM (64)	Intervention group: 500 mg/day hesperidin (*n* = 31) Control group: placebo (*n* = 29)	6 weeks	A significant decrease in TNF‐α, IL‐6, and hs‐CRP A significant increase in TAC A significant decrease in IL‐6 and hs‐CRP within hesperidin group A significant increase in TAC within hesperidin group

Abbreviations: 8‐OHDG, 8‐hydroxydeoxyguanosine; ALT, alanine aminotransferase; ApoA‐I, Apolipoprotein A‐I; apoB, Apolipoprotein B; AST, aspartate transaminase; BMI, body mass index; DBP, diastolic blood pressure; FBS, fasting blood sugar; FFM, fat‐free mass; FM, fat mass; HbA1c, hemoglobin A1c; HDL, high‐density lipoprotein; HOMA‐IR, homeostatic model assessment for insulin resistance; hs‐CRP, high‐sensitivity C‐reactive protein; ICAM‐1, intercellular adhesion molecule 1; IL‐6: interleukin‐6; LDL, low‐density lipoprotein; LMP, lifestyle modification program; MDA, malondialdehyde; NAFLD, nonalcoholic fatty liver disease; SBP, systolic blood pressure; T2DM, type 2 diabetes mellitus; TAC, total antioxidant capacity; TC, total cholesterol; TG, triglyceride; VCAM‐1, vascular cell adhesion molecule 1.

Moreover, hesperidin can inhibit the expression of genes involved in all phases of adipogenesis including peroxisome proliferator‐activated receptor (PPAR)‐γ, perilipin, sterol regulatory element‐binding protein 1 (SREBP1), fatty acid synthase, and stearoyl‐CoA desaturase (SCD; Chambers et al., 2019; Gómez‐Zorita et al., 2017). Hesperidin also significantly downregulated the expression of other genes that participated in fat metabolism such as fatty‐acid desaturase (FAT‐6 and FAT‐7), acetyl‐CoA carboxylase‐2, and SCD (Peng et al., 2016). Results of an animal study showed that hesperidin enhanced blood lipid profile, reduced hepatic lipid repletion, and ameliorated NASH in mice fed a Western diet (Mosqueda‐Solís, Sánchez, Reynés, et al., 2018).

Additionally, a clinical trial study was conducted to examine the impacts of hesperidin on NAFLD parameters, in which 1 g of hesperidin was administered to subjects with NAFLD for 3 months. The findings demonstrated a considerable reduction in serum concentration of TG, TC, and LDL‐C in the group consuming hesperidin, although only the TG and TC reduction in serum was considerably higher in the hesperidin group compared to the placebo (Cheraghpour et al., 2019). On the other hand, Haidari et al. (2015) reported that consuming 600 mg of hesperidin per day for 4 weeks significantly enhanced HDL‐C in patients with myocardial infarction, although this study did not observe a meaningful improvement in other lipid profiles. These results are incompatible with Demonty et al. (2010) study, in which consuming 800 mg hesperidin in the shape of supplementation for 4 weeks did not affect serum HDL‐C and TG concentrations in individuals with moderate hypercholesterolemia. Moreover, A meta‐analysis investigating the effects of hesperidin supplementation on lipid profile reported that hesperidin might not affect enhancing lipid profile parameters (Mohammadi et al., 2019).

## 4 HESPERIDIN AND GLUCOSE METABOLISM

Disturbances of glucose metabolism beyond a certain limit can cause NAFLD, so there is a close relationship between glucose homeostasis, fat metabolism in the liver, and NAFLD. Hesperidin can improve glucose and insulin metabolism. A randomized clinical trial study has demonstrated that consumption of hesperidin capsules (500 mg/day) for 12 weeks reduced fasting glucose levels, compared to both baseline values and the control group in patients with metabolic syndrome. Moreover, hesperidin significantly increased insulin sensitivity by assessing the homeostatic model assessment for insulin resistance (HOMA‐IR; Mohammadi et al., 2019). In a study conducted by Ribeiro et al. (2017), 500 mL orange juice consumption for 12 weeks could significantly decrease insulin levels by 18% and HOMA‐IR index by 33% in individuals with obesity. Similarly, in another study, the consumption of 300 mL of orange juice for 2 months in healthy women had a significant reduction in fasting plasma glucose and insulin levels, as well as HOMA‐IR index (Lima et al., 2019). However, the evidence provided by many clinical trial studies in various populations has shown that hesperidin did not significantly change glucose or insulin levels (Constans et al., 2015; Homayouni et al., 2017; Morand et al., 2011). This study was consistent with another study by Cheraghpour et al. (2019), who reported that 3 months of supplementation with 1‐g hesperidin significantly improved blood glucose levels compared to baseline values, although there was no significant difference between intervention and placebo groups. Another study observed that 600 mg hesperidin for 6 weeks could not affect blood glucose and insulin resistance in subjects with type 2 diabetes mellitus (Haidari et al., 2015).

Blood glucose‐lowering effects of hesperidin may be achieved by modulating the main regulatory enzymes of glucose metabolism. Hesperidin downregulated the gene expression of glucose‐6‐phosphatase, glucokinase, alpha‐ketoglutarate, and oxaloacetate and inhibited pyruvate production and hepatic gluconeogenesis, thereby affecting hyperglycemia (Akiyama et al., 2009; Jung et al., 2006). Also, in the liver of rats, hesperidin stimulates glycogenolysis and glycolysis (do Nascimento et al., 2018). Moreover, the hypoglycemic effects of hesperidin have been shown in rat models of diabetes (Franke et al., 2018). Several studies have demonstrated that hesperidin might potentially be used to regulate postprandial blood glucose by reducing the intestinal glucose transporter (Kerimi et al., 2019; Shen et al., 2012).

PPAR‐c, a nuclear receptor, regulates metabolic pathways by regulating energy homeostasis, modulating glucose and fatty acid metabolism, stimulating insulin secretion, and promoting insulin sensitivity. It is worth noting that hesperidin improves glucose metabolism by modulating PPAR‐c activation and reducing the accumulation of body and hepatic fat (Shin et al., 2013). In addition, hesperidin indirectly affects insulin resistance by modulating the composition and functionality of intestinal microbiota to induce the generation of short‐chain fatty acids, thus modulating the metabolism of lipids and inflammation, and ameliorating glucose intolerance and insulin sensitivity (Lima et al., 2019). Growing evidence suggests that hesperidin ameliorates oxidative damage and mitochondrial dysfunction, ultimately reducing insulin resistance in cells stimulated with high glucose. These changes seem to be associated with upregulated phosphorylation of AKT and glycogen synthase kinase‐3β and decreased insulin receptor substrate‐1 (IRS1; Tian et al., 2021).

Treatment of HepG2 cells with naringin and neohesperidin increased glucose uptake, which was accompanied by induced phosphorylation levels of adenosine monophosphate‐activated protein kinase (AMPK; Zhang et al., 2012). In addition, hesperidin modulates insulin resistance by regulating IRS1 and glucose transporter‐2 pathways through the downregulation of toll‐like receptor‐4 and nuclear factor‐κB (NF‐κB) expression in HepG2 cells (Xuguang et al., 2019). A study illustrated that the hypoglycemic activity and antioxidant capacity of hesperidin are due to the inhibition of oxidative stress and the formation of advanced glycation end‐products which play a critical role in the development of diabetes (Dhanya & Jayamurthy, 2020).

Overall, it seems that hesperidin can be effective in reducing insulin resistance and improving glucose metabolism, thereby improving metabolic complications in patients with NAFLD.

## 5 HESPERIDIN AND LIVER ENZYMES

Oxidative stress markers and mediators in the liver like lipid peroxidation products and liver enzymes have been proposed as indicators of NAFLD. Limited studies have investigated the impacts of hesperidin on subjects affected with NAFLD. It appears that hesperidin ameliorates the progress of NAFLD due to its impacts on the improvement of lipid peroxidation, metabolism of glucose, oxidative stress, and inflammatory pathways.

Hesperidin significantly increased the levels of glutathione peroxidase and superoxide dismutase and reduced the oxidative state in rodents fed a high‐fat diet, which may ameliorate NAFLD (Yasım et al., 2011). Despite the positive results at the preclinical level, the effects of hesperidin consumption on liver enzyme concentration in humans are not certain. The findings of a clinical trial study indicated that 12 weeks of supplementation with 1‐g hesperidin reduced significantly alanine aminotransferase and γ‐glutamyl transferase. Moreover, the results of the FibroScan showed that hepatic steatosis was meaningfully improved in the hesperidin group in comparison with the placebo group (Cheraghpour et al., 2019). Other studies have illustrated that hesperidin supplementation significantly decreased the fatty liver score, serum lipid profiles, liver injury, and hepatic steatosis (Chen et al., 2022; Cheraghpour et al., 2019). Similar to this study, Yari et al. (2021) showed that the combination of hesperidin with flaxseed caused a significant reduction in plasma levels of alanine aminotransferase, and indices of glucose metabolism and improved the grade of hepatic fibrosis.

In experimental animal research carried out by Wang et al. (2020), neohesperidin could significantly reduce hepatic steatosis and systematic insulin resistance in rodents fed a high‐fat diet. Moreover, they showed a significant enhancement in hepatocellular mitochondrial function and fatty acid oxidation with induced expression of peroxisome proliferator‐activated receptor γ coactivator‐1 (PGC‐1α). Although most of the studies focus on modification of the metabolic indicators related to fatty liver, it seems that in the limited studies conducted on the effects of hesperidin in fatty liver, it was able to reduce the process of fibrosis and hepatic steatosis.

## 6 HESPERIDIN AND INFLAMMATION

It has long been known that inflammation is regarded as one of the critical determinants contributing to the progression and pathogenesis of liver disorders. NAFLD has been identified as a low‐grade and chronic inflammation in the hepatocytes that drives systemic impacts, recognized by systemic changes in humoral factors and circulating immune cell subsets (Behrouz et al., 2021; Gehrke & Schattenberg, 2020). During NAFLD, mitochondria and β‐oxidation dysfunction decrease the hepatic PPARα activity, increase liver triglycerides and free fatty acids, suggest lipotoxicity and oxidative stress, and ultimately result in hepatic inflammation, hepatocellular apoptosis, and fibrosis (Friedman et al., 2018; Ipsen et al., 2018; Spahis et al., 2017; Takaki et al., 2013).

Excessive production of reactive species, which include reactive sulfur species, reactive nitrogen species, and reactive oxygen species (ROS), subsequently results in the secretion of various serum markers of inflammation including C‐reactive protein (CRP), interleukins (ILs), tumor necrosis factor (TNF), and other general immunity factors (Gonzalez et al., 2020; Luo & Lin, 2021). The transcription factor activation of NF‐κB signaling cascade has a key function in the immune and inflammatory responses of the body through the expression of several mediators such as inducible nitric oxide synthase, TNF‐α, cyclooxygenase‐2 (COX‐2), and IL‐6 in subjects affected with NAFLD (Chen, Xing, et al., 2020). In fact, upregulation of the NF‐κB cascade is intimately associated with the stimulation of mitogen‐activated protein kinases (MAPKs), involved in intracellular signaling cascades during pro‐inflammatory immune conditions (Parhiz et al., 2015).

A growing body of evidence has been carried out to investigate the impacts of hesperidin, its synthetic by‐products, or its metabolites on inflammatory reactions in some inflammatory‐based disorders. Experimental studies have demonstrated significant advantageous effects and mechanistic insight into hesperidin (Kumar et al., 2023). However, human studies investigating the impacts of hesperidin in subjects affected with NAFLD are still limited. A 12‐week administration of 1 g of hesperidin combined with lifestyle correction could improve NAFLD‐related risk indicators by preventing NF‐κB activation and reducing hs‐CRP and TNF‐α, compared to lifestyle correction alone in patients with NAFLD. It should be noted that the evaluation of NF‐κB p65 activity was done in peripheral blood mononuclear cells, which is one of the strengths of this study (Cheraghpour et al., 2019). Taking other metabolic disorders into account, it has been observed that taking 500 mg of hesperidin decreased circulating inflammatory molecules in individuals affected with metabolic syndrome via suppressing ORS generation (Ghanim et al., 2010). Homayouni et al. (2017) revealed that 500 mg/day hesperidin improved several inflammatory molecules such as circulating IL‐6, TNF‐α, and hs‐CRP after 6 weeks of supplementation compared to the control group in individuals affected with type 2 diabetes.

In contrast, another research found that circulating IL‐6 and hs‐CRP are not improved in subjects affected with diabetes after consuming 500 mg/day of hesperidin for 2 months. This could be due to the short duration of the intervention of 2 months (Eghtesadi et al., 2016). These findings are noteworthy because insulin resistance has a main function in the pathogenesis of NAFLD. Also, it is worth mentioning that 7‐day supplementation with 500 mL red orange juice, one of the main sources of hesperidin, had considerable anti‐inflammatory effects that led to decreasing hs‐CRP, TNF‐α, and IL‐6 concentrations in subjects with increased cardiovascular risk (Buscemi et al., 2012). However, a before‐after clinical study showed no significant alterations in circulating concentrations of CRP and IL‐6 after 4‐week supplementation with orange juice or hesperidin in healthy volunteers, which could be explained by the almost normal inflammatory levels in the healthy population (Morand et al., 2011).

In NAFLD rodent models, 12‐week supplementation with hesperidin was related to a meaningful reduction in some inflammatory molecules such as TNF‐α, IL‐6, and IL‐1β. It is believed that the suppression of endoplasmic reticulum stress‐related biomarkers in the liver immune cells prevents the activation of inflammatory pathways including NF‐κB, and consequently NAFLD development (Xie et al., 2022). Hesperidin could attenuate inflammatory and oxidative damage induced by hyperglycemia by reducing malondialdehyde, nitric oxide, and IL‐6 concentrations and improving adiponectin expression and glutathione levels in rodent models of diabetes (Mahmoud, 2013; Mahmoud et al., 2012). It can also alter oxidative damage in hepatocytes by affecting factors associated with hepatic fatty acid oxidation (Constantin et al., 2013). Moreover, hesperidin has been reported to decrease hs‐CRP, macrophage chemoattractant protein 1 (MCP‐1), and IL‐6 and increase the serum total antioxidant capacity, thereby preventing oxidative stress and inflammation caused by hyperlipidemia and hyperglycemia in rodents fed a high‐fat diet (Ferreira et al., 2016).

Considering that the majority of studies report positive effects of hesperidin on inflammation, there is still insufficient data to demonstrate the exact mechanisms of action. Nonetheless, it seems that hesperidin, as an antioxidant, inhibits ROS production that potentially interferes with the activation of transcription factors like NF‐κB and nuclear translocation by blocking the phosphorylation of NF‐κβ inhibitor (IκB). Additionally, hesperidin inhibits the phosphorylation of MAPKs, extracellular signal‐regulated kinase (ERK), and a lesser degree C‐jun N‐terminal kinase (JNK), which are important signaling pathways in the inflammatory processes. Other potential mechanism includes the upregulation of PPARγ, a nuclear transcription factor involved in inhibiting NF‐κB activation and the production of different inflammatory agents, chemokines, and adhesion molecules (Cheraghpour et al., 2019; Tejada et al., 2018). Cyclooxygenase‐1 and cyclooxygenase‐2 are the main enzymes for the production of pro‐inflammatory prostaglandins from arachidonic acid, which in turn contribute to inflammatory responses. Studies highlighted that hesperidin can suppress cyclooxygenase‐2 gene expression and block the generation of prostaglandins leading to the inhibition of inflammatory markers (Hirata et al., 2005; Lawrence, 2009; Vabeiryureilai et al., 2015).

## 7 HESPERIDIN AND BODY COMPOSITION

The major objective of NAFLD control is to achieve at least a 5% reduction in body weight (Chalasani et al., 2012). Multiple lines of evidence report that hesperidin has anti‐obesity activity. It can induce the secretion of cholecystokinin, an anorexigenic gut hormone, in enteroendocrine (Kim et al., 2013). It has been suggested that dietary bioflavonoid hesperidin may induce its anti‐obesity effects partially via the prevention of hepatic lipogenesis (Ohara et al., 2015). Citrus flavonoids like hesperidin have been found to induce the browning of white adipocytes, promote energy balance and thermogenesis, and reduce plasma lipids concentrations and obesity through an AMPK‐mediated pathway (Mosqueda‐Solís, Sánchez, Portillo, et al., 2018; Xiong et al., 2019; Zhang et al., 2019).

In addition, the beneficial effect of hesperidin treatment on obesity was related to increased expression of uncoupling protein 3, which in turn improves energy expenditure from lipids (Kim et al., 2019). Accumulating data demonstrated that hesperidin has positive effects on lipid accumulation and adiposity (Kim et al., 2019; Mosqueda‐Solís et al., 2017; Serino & Salazar, 2018). Several animal models of obesity or metabolic syndrome have reported a body‐weight‐reducing effect in response to hesperidin supplementation (Mayneris‐Perxachs et al., 2019; Pu, 2016; Sun et al., 2017; Wu et al., 2017), as well as a reduction in adiposity (Mayneris‐Perxachs et al., 2019; Pu, 2016; Wang et al., 2011; Wu et al., 2017). For example, in a rodent model, hesperidin supplementation decreased body weight, fat mass, and plasma lipids in rodents fed a high‐fat diet. This effect is mediated by improving intestinal barrier integrity and modulating the composition of intestinal microbiota (Liu et al., 2020). In contrast, daily hesperidin supplementation (100 mg/kg body weight) for 2 months had no meaningful alterations in the body weight of rodents fed a Western diet, although hesperidin was able to reduce adipocyte size (Mosqueda‐Solís, Sánchez, Reynés, et al., 2018).

In a clinical trial, a daily intake of 500 mg α‐glucosyl hesperidin, the soluble hesperidin derivative, reduced abdominal fat significantly in individuals with a moderately high body mass index compared to baseline after 12 weeks of supplementation. However, this reduction was not significantly different from those of subjects taking a placebo (Hanawa et al., 2008). These findings are similar to Aptekmann et al. study which reported a significant body weight reduction after daily use of orange juice for 13 weeks in individuals with hypercholesterolemia, without significant change between the intervention and control groups (Aptekmann & Cesar, 2010). Also, other human clinical trials revealed that 500 mg α‐glucosyl hesperidin did not exert anti‐obesity effects in individuals with moderate obesity (Ohara et al., 2015). This result is consistent with the Yari et al. (2021) study, in which hesperidin supplementation failed to induce weight‐loss effects. Overall, hesperidin or orange juice supplementation in subjects with overweight or obesity does not reveal the beneficial effects observed in animal models.

## 8 LIMITATIONS AND STRENGTHS

To the best of our knowledge, this is the first review study demonstrating the potential effects of hesperidin on NAFLD parameters. Our review also discussed the mechanisms of actions of hesperidin on several aspects of NAFLD that may be less well‐regarded. The lack of sufficient clinical data on the therapeutic effects of hesperidin is an important limitation that can be mentioned in most of the previous studies. Therefore, clinical studies, especially those focused on the appropriate dosage, bioavailability, efficacy, and safety of hesperidin and its metabolites, are warranted before extending flavonoid therapy to humans.

## 9 CONCLUSION

**FIGURE 1:**
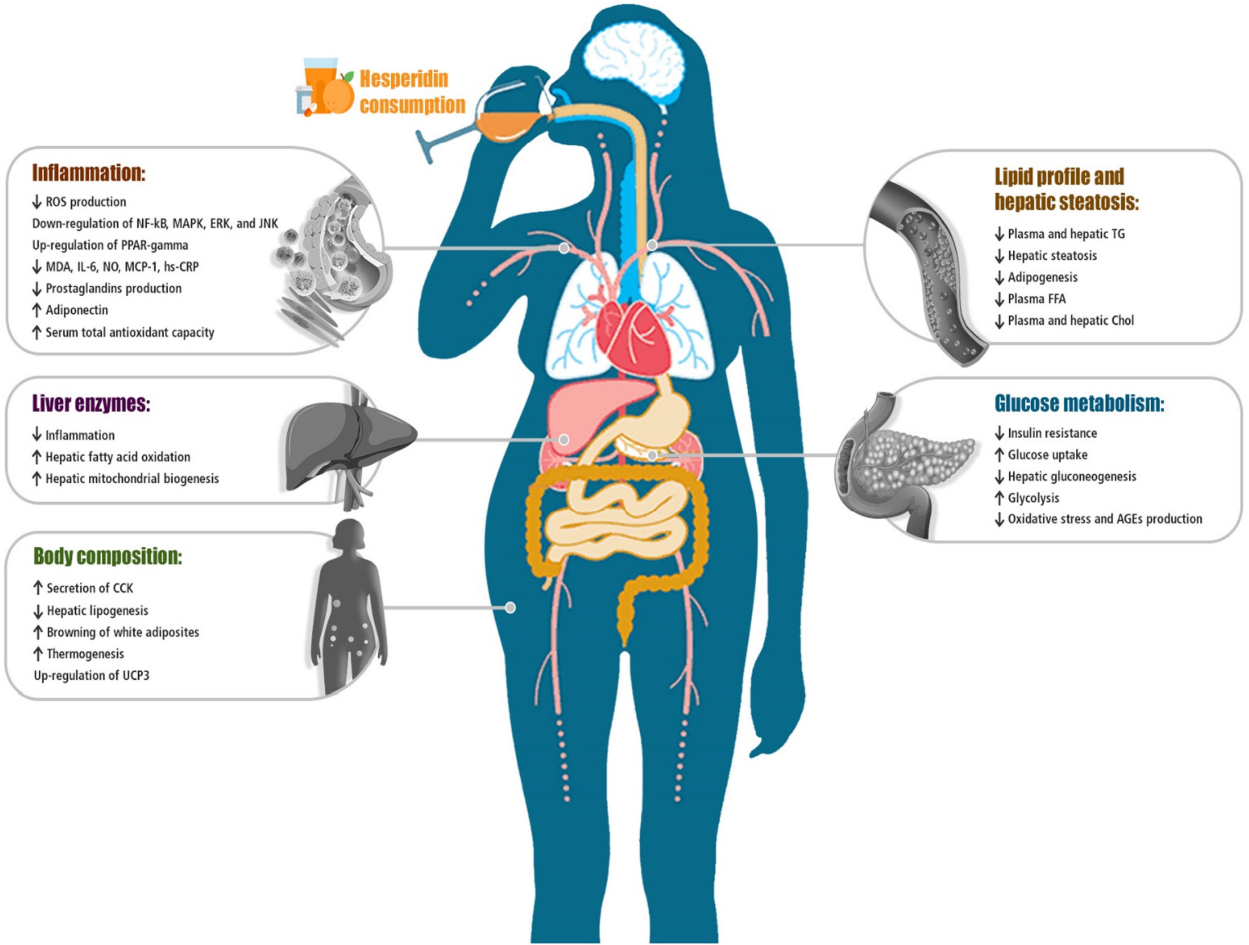
Summary of the most important effects of hesperidin consumption on proposed mechanisms of action in NAFLD. CCK, cholecystokinin; CRP, C‐reactive protein; IL‐6, interleukin‐6; MCP‐1, monocyte chemoattractant protein‐1; MDA, malondialdehyde; NO, nitric oxide; PPAR, peroxisome proliferator‐activated receptors; ROS, reactive oxygen species; TG, triglyceride; UCP3, uncoupling protein 3.

The NAFLD represents a significant burden of disease worldwide, which is alarmingly worsening each year. The important pathophysiological mechanisms underlying NAFLD are several including oxidative stress, inflammation, liver fibrosis, and apoptosis. Currently, therapeutic approaches are not ideal for managing NAFLD, thus new approaches and treatments are still needed. Hesperidin has a remarkable effect on hepatocytes and brings new hope to people with NAFLD around the world. Primary evidence has suggested a reverse association between hesperidin and NAFLD risk factors including oxidative stress, inflammation, dyslipidemia, hyperglycemia, and obesity (Figure 1). Animal studies have demonstrated that taking hesperidin could decrease NAFLD severity. Accordingly, it was expected that the use of hesperidin might improve NAFLD. Overall, human studies investigating the impacts of hesperidin on NAFLD are rare. It has been indicated that hesperidin might contribute to the improvement of NAFLD through glucose‐lowering effect, alterations in liver fat content and lipid profile, and anti‐inflammatory and antioxidant properties. Due to the limited number and heterogeneity of existing human studies, further research is required to confirm these results in human beings.

## AUTHOR CONTRIBUTIONS

**Nava Morshedzadeh:** Conceptualization (equal); investigation (equal); methodology (equal); writing – original draft (equal). **Amirhossein Ramezani:** Data curation (equal); investigation (equal); writing – original draft (equal). **Vahideh Behrouz:** Investigation (equal); supervision (equal); writing – original draft (equal); writing – review and editing (equal). **Elias Mir:** Software (lead).

## FUNDING INFORMATION

No external funding was received to support this work.

## CONFLICT OF INTEREST STATEMENT

The authors declare that they have no competing interests.

## Data Availability

The data that support the findings of this study are available on request from the corresponding author. The data are not publicly available due to privacy or ethical restrictions.
